# Quantitative Assessment and Diagnosis for Regional Agricultural Drought Resilience Based on Set Pair Analysis and Connection Entropy

**DOI:** 10.3390/e21040373

**Published:** 2019-04-05

**Authors:** Menglu Chen, Shaowei Ning, Yi Cui, Juliang Jin, Yuliang Zhou, Chengguo Wu

**Affiliations:** 1 School of Civil Engineering, Hefei University of Technology, Hefei 230009, China; 2 State Key Laboratory of Hydraulic Engineering Simulation and Safety, Tianjin University, Tianjin 300072, China

**Keywords:** regional agricultural drought resilience, assessment and diagnosis, vulnerability index, connection entropy, set pair potential, Huaibei Plain

## Abstract

Assessment and diagnosis of regional agricultural drought resilience (RADR) is an important groundwork to identify the shortcomings of regional agriculture to resist drought disasters accurately. In order to quantitatively assess the capacity of regional agriculture system to reduce losses from drought disasters under complex conditions and to identify vulnerability indexes, an assessment and diagnosis model for RADR was established. Firstly, this model used the improved fuzzy analytic hierarchy process to determine the index weights, then proposed an assessment method based on connection number and an improved connection entropy. Furthermore, the set pair potential based on subtraction was used to diagnose the vulnerability indexes. In addition, a practical application had been carried out in the region of the Huaibei Plain in Anhui Province. The evaluation results showed that the RADR in this area from 2005 to 2014 as a whole was in a relatively weak situation. However, the average grade values had decreased from 3.144 to 2.790 during these 10 years and the RADR had an enhanced tendency. Moreover, the possibility of RADR enhancement for six cities in this region decreased from east to west, and the drought emergency condition was the weak link of the RADR in the Huaibei Plain.

## 1. Introduction

Drought is a natural phenomenon, which usually arises from a severe shortage of regional water resources over a certain period of time [1]. It is also a recurring pattern of climate that occurs within nearly all climatic regions, which can be exacerbated by the imbalance between water supply and water demand over time [2,3]. With the occurrence of severe droughts around the world, various problems caused by them have aroused great attention from all walks of life [4]. These complex phenomena include water scarcity and water shortage [5,6], indicating that drought may have a more profound impact on human society than other natural disasters. Statistics show that about 1/3 of the world’s lands and 1/5 of the population are threatened by droughts every year [7]. Across many regions, more extreme droughts are expected in the twenty-first century due to the combined effects of global climate change and precipitation anomaly. Indeed, all kinds of losses caused by droughts will increase in the coming period [8,9]. Therefore, the research on drought has become the focus of water resources and natural hazards studies.

In recent years, drought has induced a complex series of effects that involve many sectors of human society, including the social economy and residents living [10]. Many scholars have brought plenty of research on drought identification, drought monitoring, and drought risk assessment, but the research on regional drought resilience is still in the primary development stage around the world. In the USA, the National Drought Resilience Partnership (NDRP) established in 2013 was designed to use existing programs in a concerted effort to help communities, especially for the agricultural community to be better prepared in the future [11,12].

In addition, the drought in the south-east of England from 2004–2006 created important insights for debates in water management. They suggested a water resources management system based on the cap and trade concept to initiate debates for water shortage parts in England, thus as to deliver resistance to drought [13]. Because droughts affect all aspects of human social and economic life, the regional drought resilience also includes different types of drought-resistant objects: (1) Social ecosystem: The quantitative model of drought pressure and drought resilience analyzes the variation trend of the social-ecological system’s drought pressure and resilience for the period from 2000 to 2010 in Henan Province [14]. A unique multivariate approach was proposed as a measure of socioeconomic drought, termed Multivariate Standardized Reliability and Resilience Index (MSRRI) [15]; (2) Populations: Populations crashed most severely in drier regions but the landscape structure around sites influenced population responses as well. Larger patches of woodland habitat reduced population sensitivity to the drought event and also facilitated faster recovery [16]. The application of Bayesian networks (BNs) to drought resilience and household livelihood provides evidence that watershed development and management has influenced household resilience to drought [17]; (3) Water supply: Long severe droughts in the nineteenth century provided an opportunity to test water supply system behavior in a range of actual drought events. The test indicates that significant demand restrictions and engineering measures had to be introduced [18]. The framework to analyze dynamics in the governance system for urban water service applied to a drought-prone city in central Vietnam finds that changes and systematic interventions are needed to enhance resilience [19]; (4) Agriculture: Research shows that the capacity of land users to cope with drought is influenced by the ago-ecosystems resilience, the diversity of livelihood options, access to resources and institutional support [20]. Moreover, farmers’ response mechanisms have evolved, expanding from short-term adjustments to long-term adaptations [21]. Since major challenges are to seek ways of mitigating and coping with droughts, building agricultural resilience in farming systems is also a means of water resources management [22]. The importance of adopting drought management approach in the farming sector is shown to be crucial for improving decision-making in future drought events [23]. Actually, the agriculture drought resilience strategies evolve over time with the ecosystem management support to build social-ecological resilience to droughts [24]. In 2005, a mathematical clustering method has been used to evaluate the ability to cope with agricultural drought for 31 provinces in China, the result is significant background data for drought management [25].

Compared with other industrial sectors, agricultural production is more vulnerable to the adverse effects of droughts under the environment of climate change [26]. The regional agricultural drought resilience (RADR) has become the key link to regional drought resilience. As a buffering effect, the resilience gives the regional agricultural system the ability to promote the disaster-bearing body to constantly improve the ability to cope with external disaster-causing factors, thus as to mitigate the losses caused by various shocks. Therefore, the research about RADR is a crucial basic work to carry out regional drought prevention and disaster reduction measures [27,28]. The quantitative assessment and diagnosis of RADR are vital to identify the shortcomings and clarify the key direction in the future agricultural drought risk management.

Based on the current research on drought risk system, the RADR should include both the natural and social attributes, it is a fundamental work for regional agricultural drought risk control. However, the systematic theory and quantified method of RADR have not been completely formed. In addition, an applicable diagnosis model of RADR has not been proposed to identify the vulnerable factors for improving drought resilience. Therefore, this study provides a comprehensive assessment of RADR based on the theoretical analysis of natural disaster risk system across the Huaibei Plain, China. Regional Agricultural Drought Resilience Assessment Index (RADRAI) system is built for each city with the corresponding evaluation grade standards from the perspective of system theory. Furthermore, a quantitative assessment and diagnosis model of RADR was established based on a set pair analysis method and entropy theory. This study attempts to answer the following issues:

- Identifying the main influence factors for quantifying the RADR in the Huaibei Plain.
- Establishing an evaluation index system and determining the weights for RADR.
- Evaluating the RADR for each city in the Huaibei Plain for the period from 2005 to 2014.
- Assessing the situations of RADR and the stability for each city in the Huaibei Plain.
- Analyzing the driving mechanism and diagnosing the main factors for weakening the RADR.

## 2. Construction of RADRAI

### 2.1. Influence Factors

The first step for evaluating RADR is to identify the factors that influence the dimension of drought, including environment, health, society, and economy [29]. Correspondingly, the definitions of RADR can be divided into two categories. One is from the view of human activities, defining the RADR as the capability of human beings to protect crop yield from drought during the crop growth period in a given region [25]. Another is from the perspective of the disaster level, defining the RADR as the level of resistance to a given degree of drought for guaranteeing agricultural production [30].

In terms of influence factors, natural factors include the climatic characteristics, water resources conditions, topography, geomorphology, soil types, crop species, and crop growth periods. In this study, the influence factors of RADR were divided into two main aspects including the natural environment and anthropogenic activities. Since the impacts on nature and society are distinct for different areas, relevant factors should be selected based on expert knowledge and experiences about the study area.

### 2.2. Construction of RADRAI

Based on the analysis of the concept and influencing factors of RADR, adding up with the principles for constructing the evaluation index system, the index system for evaluating RADR was divided into five subsystems: Regional natural condition, water conservancy condition, economic and social conditions, scientific and technological conditions, and drought emergency condition [28,31]. According to the characteristic of each evaluation subsystem, they were further decomposed into 14 evaluation indexes (Table 1). The RADRAI consisted of five subsystems and the corresponding 14 indexes, which could be described as {*x* (*k*, *j*) |*k* = 1, 2, 3, 4, 5; *j* = 1, 2, …, *n_k_*} represents the index *j* in the subsystem *k* in an evaluation region, *n_k_* is the number of indexes in the subsystem *k*.

Based on the actual meaning of the RADRAI, the statistical characteristics and the comprehensive function analysis of these indexes, the evaluation grade standard was established. To be simple and general, the level of each index in RADRAI was divided into five-grade standards, which were grade I (very strong), grade II (strong), grade III (moderate), grade IV (weak) and, grade V (very weak). To sum up, the sample data set of RADRAI was recorded as {*x* (*i*, *k*, *j*) | *k* = 1, 2, 3, 4, 5; *i* = 1, 2, …, *N*; *j* = 1, 2, …, *n_k_*}, where *N* is the total number of all evaluation regions. The grade standards for each index were described as {*s* (*p*, *j*) |*p* = 1, 2, 3, 4, 5; *j* = 1, 2, …, *n_k_*}, where *p* represents each degree of RADR.

## 3. Methodology

The assessment and diagnosis model of RADR in this study was established by the following seven steps:

Step 1: Determining the weight of each evaluation index in RADRAI.

The weight of each index was calculated based on the information from the decision makers. In this study, the Improved Fuzzy Analytic Hierarchy Process based on Accelerated Genetic Algorithm (AGA-FAHP) [32] was used to determine the index weight in RADRAI, expressed as {*w*_s_ (*k*, *j*)|*k* = 1, 2, 3, 4, 5; *j* = 1, 2, …, *n_k_*}, representing the weight of the evaluation index *j* in the subsystem *k*. The distribution in a judgement matrix resulted in a fuzzy reciprocal square matrix named Fuzzy Consistency Judgment Matrix (FCJM), represented as:

(1) $$A^{k}=\left(a_{jl}^{k}\right)=\left(\begin{array}{cccc} a_{11} & a_{12} & \ldots & a_{1n_{k}}\\ a_{21} & a_{22} & \ldots & a_{2n_{k}}\\ \ldots & \ldots & \ldots & \ldots \\ a_{n_{k}1} & a_{n_{k}2} & \ldots & a_{n_{k}n_{k}} \end{array}\right)$$

where *a^k^_jl_* (*k* = 1, 2, 3, 4, 5; *j* = 1, 2, …, *n_k_*; *l* = 1, 2, …, *n_k_*) indicates the degree to which the index *j* is more important than the index *l* in the subsystem *k*, 0 ≤ *a^k^_jl_* ≤ 1, *a^k^_jl +_ a^k^_lj_* = 1.

When *a^k^_jl_* = 0.5, it meant that the index *j* was as important as the index *l*, meanwhile, when *a^k^_jl_* > 0.5, it meant that the index *j* was more important than the index *l*. *a^k^_jl_* could be calculated using the following equations:

(2) $$a_{jl}^{k}={\hat{y}}_{l}^{k}/\left({\hat{y}}_{j}^{k}+{\hat{y}}_{l}^{k}\right)$$

(3) $${\hat{y}}_{l}^{k}=\overset{n_{a}}{\underset{a=1}{\sum }}y_{a,k,l}/n_{a}$$

where *y_a, k, l_* indicates the importance sort value of the index *l* in the subsystem *k* judged by the expert *a*, then *a* = 1, 2, …, *n_a_*. *n_a_* is the total number of experts.

Using the AGA-FAHP method to adjust the consistency of *A^k^* and calculate the weight *w*_s_ (*k*, *j*) of each index. If the *A^k^* is completely consistent, then [33,34]:

(4) $$\overset{n_{k}}{\underset{j=1}{\sum }}\overset{n_{k}}{\underset{l=1}{\sum }}\left|0.5\left(n_{k}-1\right)\left[w_{s}\left(k,j\right)-w_{s}\left(k,l\right)\right]+0.5-a_{jl}^{k}\right|/n_{k}^{2}=0$$

where the left item is the consistency index of *A^k^*. If the consistency index of *A^k^* is not greater than a critical value, then the matrix *A^k^* has a satisfactory consistency. Otherwise, it needs to be further corrected. The sorting weights of each element in the corrected matrix *B^k^* = (*b^k^_jl_*)*_n__k_*_×*n*_*_k_* were still recorded as {*w*_s_ (*k*, *j*)|*k* = 1, 2, 3, 4, 5; *j* = 1, 2, …, *n_k_*}, then the *B^k^* met the following equations [32]:

(5) $$\min CIC(n_{k})=\sum_{j=1}^{n_{k}}\sum_{l=1}^{n_{k}}\left|b_{jl}^{k}-a_{jl}^{k}\right|/n_{k}^{2}+\sum_{j=1}^{n_{k}}\sum_{l=1}^{n_{k}}\left|0.5(n_{k}-1)\left[w_{s}(k,j)-w_{s}(k,l)\right]+0.5-a_{jl}^{k}\right|/n_{k}^{2}=0$$

(6) $$s.t.\;\{\begin{array}{l} b_{jj}^{k}=0.5\\ 1-b_{lj}^{k}=b_{jl}^{k}\in \left[a_{jl}^{k}-d,a_{jl}^{k}+d\right]\cap \left[0,1\right],(l=j+1,j+2,\ldots ,n_{k})\\ \sum_{j=1}^{n_{k}}w_{s}(k,j)=1\\ w_{s}(k,j)>0 \end{array}$$

where the *CIC* (*n_k_*) is the consistency index coefficient, *d* is the non-negative parameter, according to the author’s experience, it can be selected from [0, 0.5]. The number of optimization variables in Equation (5) is *n_k_* (*n_k_* + 1)/2.

Obviously, the smaller the value of *CIC* (*n_k_*) is, the higher the consistency of FCJM. When the value of *CIC* (*n_k_*) is less than a critical value, it can be considered that *A^k^* has a satisfactory consistency, and the obtained index weights are acceptable. After a lot of numerical calculation and referring to the relevant literature [33,34], the matrix can be considered to have a satisfactory consistency when the value of *CIC* (*n_k_*) is less than 0.20.

Step 2: Establishing the five-elements connection number of evaluation sample.

Set Pair Analysis (SPA) was a new systematic analysis method proposed by Zhao (1989) [35] to quantitatively deal with the uncertain problems in a certain-uncertain system. For two given sets *A* and *B*, assuming *M* was the total number, *S* represented the identity features, *P* represented the contrary features, *F* represented the discrepancy features, then the Connection Number (CN) between *A* and *B* (*u_A–B_*) was defined as follows [36]:

(7) $$u_{A–B}=S/M+\left(F/M\right)I+\left(P/M\right)J$$

where *S* + *F* + *P* = *M*, there were the ratios *S*/*M* = *a, F*/*M* = *b, P*/*M* = *c*. *I* is the difference coefficient and *J* is the opposition coefficient. Then Equation (6) becomes:

(8) μ=a+bI+cJ

Equation (7) is the basic expression of the Three-Elements Connection Number. If the uncertain term *b* is decomposed into three parts and applied to the assessment of the RADR in a given evaluation area *i*, the establishment of the five-elements connection number of evaluation sample *u*_1*i*_ based on Equation (7) was obtained:

(9) $$u_{1i}=a_{1i}+b_{1i}^{1}I_{1}+b_{1i}^{2}I_{2}+b_{1i}^{3}I_{3}+c_{1i}J$$

where *u*_1*i*_ is the five-elements connection number of evaluation sample (SFECN) of RADR in the evaluation area. $a_{1i},b_{1i}^{1},b_{2i}^{2},b_{3i}^{3},c_{1i}$ are the connection number components, $a_{1i},b_{1i}^{1},b_{2i}^{2},b_{3i}^{3},c_{1i}\in [0,1]$ and $a_{1i}+b_{1i}^{1}+b_{2i}^{2}+b_{3i}^{3}+c_{1i}=1$, *a*_1*i*_ is the identity degree, $b_{1i}^{1},b_{2i}^{2},b_{3i}^{3}$ are the discrepancy degrees, *c*_1*i*_ is the contrary degree. *I*_1_, *I*_2_, *I*_3_ are the difference coefficients [35]. *J* is the opposition coefficient, which is generally equal to −1 [31]. The five connection number components of *u*_1*i*_ can be calculated by the following equations:

(10) $$a_{1i}=\sum_{k=1}^{5}a_{1}(i,k),\;b_{1i}^{1}=\sum_{k=1}^{5}b_{1}^{1}(i,k),\;b_{1i}^{2}=\sum_{k=1}^{5}b_{1}^{2}(i,k),\;b_{1i}^{3}=\sum_{k=1}^{5}b_{1}^{3}(i,k),\;c_{1i}=\sum_{k=1}^{5}c_{1}(i,k)$$

where *a*_1_ (*i*, *k*), $b_{1}^{1}(i,k)$, $b_{1}^{2}(i,k)$, $b_{1}^{3}(i,k)$, $c_{1}(i,k)$, $a_{1}(i,k),b_{1}^{1}(i,k),b_{1}^{2}(i,k),b_{1}^{3}(i,k),c_{1}(i,k)$ are the connection number components in the subsystem *k* of the evaluation area *i*, they can be calculated by the following equations:

(11) $$\begin{array}{l} a_{1}(i,k)=\sum_{j=1}^{n_{k1}}w_{s}(i,j,k),\;b_{1}^{1}(i,k)=\sum_{j=n_{k1}+1}^{n_{k1}+n_{k2}}w_{s}(i,j,k),\;b_{1}^{2}(i,k)=\sum_{j=n_{k1}+n_{k2}+1}^{n_{k1}+n_{k2}+n_{k3}}w_{s}(i,j,k),\;\\ b_{1}^{3}(i,k)=\sum_{j=n_{k1}+n_{k2}+n_{k3}+1}^{n_{k1}+n_{k2}+n_{k3}+n_{k4}}w_{s}(i,j,k),\;c_{1}(i,k)=\sum_{j=n_{k1}+n_{k2}+n_{k3}+n_{k4}+1}^{n_{k1}+n_{k2}+n_{k3}+n_{k4}+n_{k5}}w_{s}(i,j,k) \end{array}$$

where *n_k_*_1_, *n_k_*_2_, *n_k_*_3_, *n_k_*_4_, *n_k_*_5_ are the number of indexes which belong to each evaluation grade in the subsystem *k* in the evaluation area *i*, respectively. Since the total number of indexes in the evaluation system was *n*.

Step 3: Establishing the five-elements connection number of evaluation index.

Using SPA to establish the connection number *u*_2*i*_ between the sample value of the evaluation index *j* and the evaluation grade criteria for RADR in subsystem *k* in the evaluation area *i*:

(12) $$u_{2i}=a_{2i}+b_{2i}^{1}I_{1}+b_{2i}^{2}I_{2}+b_{2i}^{3}I_{3}+c_{2i}J$$

The components in Equation (11) are the same as those in Equation (8). Similarly, the five components of *u*_2*i*_ can be calculated by the following equations:

(13) $$a_{2i}=\sum_{k=1}^{5}a_{2}(i,k),\;b_{2i}^{1}=\sum_{k=1}^{5}b_{2}^{1}(i,k),\;b_{2i}^{2}=\sum_{k=1}^{5}b_{2}^{2}(i,k),\;b_{2i}^{3}=\sum_{k=1}^{5}b_{2}^{3}(i,k),\;c_{2i}=\sum_{k=1}^{5}c_{2}(i,k)$$

where *a*_2_ (*i*, *k*), $b_{2}^{1}(i,k)$, $b_{2}^{2}(i,k)$, $b_{2}^{3}(i,k)$, $c_{2}(i,k)$, $a_{2}(i,k),b_{2}^{1}(i,k),b_{2}^{2}(i,k),b_{2}^{3}(i,k),c_{2}(i,k)$ can be calculated by the following equations:

(14) $$\begin{array}{l} a_{2}(i,k)=\sum_{j=1}^{n_{k}}a_{2}(i,j,k)\times w_{s}(i,j,k),\;b_{2}^{1}(i,k)=\sum_{j=1}^{n_{k}}b_{2}^{1}(i,j,k)\times w_{s}(i,j,k),\;b_{2}^{2}(i,k)=\sum_{j=1}^{n_{k}}b_{2}^{2}(i,j,k)\times w_{s}(i,j,k),\;\\ b_{2}^{3}(i,k)=\sum_{j=1}^{n_{k}}b_{2}^{3}(i,j,k)\times w_{s}(i,j,k),\;c_{2}(i,k)=\sum_{j=1}^{n_{k}}c_{2}(i,j,k)\times w_{s}(i,j,k) \end{array}$$

where *a*_2_ (*i*, *j*, *k*), $b_{2}^{1}(i,j,k)$, $b_{2}^{2}(i,j,k)$, $b_{2}^{3}(i,j,k)$, *c*_2_ (*i*, *j*, *k*) $a_{2}(i,j,k),b_{2}^{1}(i,j,k),b_{2}^{2}(i,j,k),b_{2}^{3}(i,j,k),c_{2}(i,j,k)$ were calculated as follows [32]:

(15) $$a_{2}(i,j,k)=\left\{ \begin{array}{l} 1,\;\mathrm{PI}:x(i,j,k)\leq s_{1}(j,k)\;or\;\mathrm{NI}:x(i,j,k)\geq s_{1}(j,k)\\ 1-\frac{2(x(i,j,k)-s_{1}(j,k))}{s_{2}(j,k)-s_{1}(j,k)},\;\mathrm{PI}:s_{1}(j,k)<x(i,j,k)\leq s_{2}(j,k)\;or\;\mathrm{NI}:s_{1}(j,k)>x(i,j,k)\geq s_{2}(j,k)\\ -1,\;\mathrm{PI}:x(i,j,k)\geq s_{2}(j,k)\;or\;\mathrm{NI}:x(i,j,k)<s_{2}(j,k) \end{array}\right.$$

(16) $$b_{2}^{1}(i,j,k)=\left\{ \begin{array}{l} 1-\frac{2(s_{1}(j,k)-x(i,j,k))}{s_{1}(j,k)-s_{0}(j,k)},\;\mathrm{PI}:x(i,j,k)\leq s_{1}(j,k)\;or\;\mathrm{NI}:x(i,j,k)\geq s_{1}(j,k)\\ 1,\;\mathrm{PI}:s_{1}(j,k)<x(i,j,k)\leq s_{2}(j,k)\;or\;\mathrm{NI}:s_{1}(j,k)>x(i,j,k)\geq s_{2}(j,k)\\ 1-\frac{2(x(i,j,k)-s_{2}(j,k))}{s_{3}(j,k)-s_{2}(j,k)},\;\mathrm{PI}:s_{2}(j,k)<x(i,j,k)\leq s_{3}(j,k)\;or\;\mathrm{NI}:s_{2}(j,k)>x(i,j,k)\geq s_{3}(j,k)\\ -1,\;\mathrm{PI}:x(i,j,k)>s_{3}(j,k)\;or\;\mathrm{NI}:x(i,j,k)<s_{1}(j,k) \end{array}\right.$$

(17) $$b_{2}^{2}(i,j,k)=\left\{ \begin{array}{l} -1,\;\mathrm{PI}:x(i,j,k)\leq s_{1}(j,k)\;or\;\mathrm{NI}:x(i,j,k)\geq s_{1}(j,k)\\ 1-\frac{2(s_{2}(j,k)-x(i,j,k))}{s_{2}(j,k)-s_{1}(j,k)},\;\mathrm{PI}:s_{1}(j,k)<x(i,j,k)\leq s_{2}(j,k)\;or\;\mathrm{NI}:s_{1}(j,k)>x(i,j,k)\geq s_{2}(j,k)\\ 1,\;\mathrm{PI}:s_{2}(j,k)<x(i,j,k)\leq s_{3}(j,k)\;or\;\mathrm{NI}:s_{2}(j,k)>x(i,j,k)\geq s_{3}(j,k)\\ 1-\frac{2(x(i,j,k)-s_{3}(j,k))}{s_{4}(j,k)-s_{3}(j,k)},\;\mathrm{PI}:s_{3}(j,k)<x(i,j,k)\leq s_{4}(j,k)\;or\;\mathrm{NI}:s_{3}(j,k)>x(i,j,k)\geq s_{4}(j,k)\\ -1,\;\mathrm{PI}:x(i,j,k)>s_{4}(j,k)\;or\;\mathrm{NI}:x(i,j,k)<s_{4}(j,k) \end{array}\right.$$

(18) $$b_{2}^{3}(i,j,k)=\left\{ \begin{array}{l} -1,\;\mathrm{PI}:x(i,j,k)\leq s_{2}(j,k)\;or\;\mathrm{NI}:x(i,j,k)\geq s_{2}(j,k)\\ 1-\frac{2(s_{3}(j,k)-x(i,j,k))}{s_{3}(j,k)-s_{2}(j,k)},\;\mathrm{PI}:s_{2}(j,k)<x(i,j,k)\leq s_{3}(j,k)\;or\;\mathrm{NI}:s_{2}(j,k)>x(i,j,k)\geq s_{3}(j,k)\\ 1,\;\mathrm{PI}:s_{3}(j,k)<x(i,j,k)\leq s_{4}(j,k)\;or\;\mathrm{NI}:s_{3}(j,k)>x(i,j,k)\geq s_{4}(j,k)\\ 1-\frac{2(x(i,j,k)-s_{4}(j,k))}{s_{5}(j,k)-s_{4}(j,k)},\;\mathrm{PI}:s_{4}(j,k)<x(i,j,k)\leq s_{5}(j,k)\;or\;\mathrm{NI}:s_{4}(j,k)>x(i,j,k)\geq s_{5}(j,k) \end{array}\right.$$

(19) $$c_{2}(i,j,k)=\left\{ \begin{array}{l} -1,\;\mathrm{PI}:x(i,j,k)\leq s_{3}(j,k)\;or\;\mathrm{NI}:x(i,j,k)\geq s_{3}(j,k)\\ 1-\frac{2(s_{4}(j,k)-x(i,j,k))}{s_{4}(j,k)-s_{3}(j,k)},\;\mathrm{PI}:s_{3}(j,k)<x(i,j,k)\leq s_{4}(j,k)\;or\;\mathrm{NI}:s_{3}(j,k)>x(i,j,k)\geq s_{4}(j,k)\\ 1,\;\mathrm{PI}:s_{4}(j,k)<x(i,j,k)\leq s_{5}(j,k)\;or\;\mathrm{NI}:s_{4}(j,k)>x(i,j,k)\geq s_{5}(j,k) \end{array}\right.$$

where the evaluation index is in accordance, it is called the Positive Index (PI), on the contrary, the index is called the Negative Index (NI). *s*_1_ (*j*, *k*)~*s*_5_ (*j*, *k*) are the critical values of the evaluation grade of the index *j* in the subsystem *k*.

Step 4: Establishing the average five-elements connection number.

Based on Equations (8) and (11), the average five-elements connection number *u_i_* of the RADR in the evaluation area *i* can be obtained by the following equations. According to the principle of minimum relative entropy [37], there were

(20) $$u_{i}=a_{i}+b_{i}^{1}I_{1}+b_{i}^{2}I_{2}+b_{i}^{3}I_{3}+c_{i}J$$

(21) $$\begin{array}{l} a_{i}=\frac{{\left(a_{1i}a_{2i}\right)}^{0.5}}{{\left(a_{1i}a_{2i}\right)}^{0.5}+{\left(b_{1i}^{1}b_{2i}^{1}\right)}^{0.5}+{\left(b_{1i}^{2}b_{2i}^{2}\right)}^{0.5}+{\left(b_{1i}^{3}b_{2i}^{3}\right)}^{0.5}+{\left(c_{1i}c_{2i}\right)}^{0.5}},\\ b_{i}^{1}=\frac{{\left(b_{1i}^{1}b_{2i}^{1}\right)}^{0.5}}{{\left(a_{1i}a_{2i}\right)}^{0.5}+{\left(b_{1i}^{1}b_{2i}^{1}\right)}^{0.5}+{\left(b_{1i}^{2}b_{2i}^{2}\right)}^{0.5}+{\left(b_{1i}^{3}b_{2i}^{3}\right)}^{0.5}+{\left(c_{1i}c_{2i}\right)}^{0.5}},\\ b_{i}^{2}=\frac{{\left(b_{1i}^{2}b_{2i}^{2}\right)}^{0.5}}{{\left(a_{1i}a_{2i}\right)}^{0.5}+{\left(b_{1i}^{1}b_{2i}^{1}\right)}^{0.5}+{\left(b_{1i}^{2}b_{2i}^{2}\right)}^{0.5}+{\left(b_{1i}^{3}b_{2i}^{3}\right)}^{0.5}+{\left(c_{1i}c_{2i}\right)}^{0.5}},\\ b_{i}^{3}=\frac{{\left(b_{1i}^{3}b_{2i}^{3}\right)}^{0.5}}{{\left(a_{1i}a_{2i}\right)}^{0.5}+{\left(b_{1i}^{1}b_{2i}^{1}\right)}^{0.5}+{\left(b_{1i}^{2}b_{2i}^{2}\right)}^{0.5}+{\left(b_{1i}^{3}b_{2i}^{3}\right)}^{0.5}+{\left(c_{1i}c_{2i}\right)}^{0.5}},\\ c_{i}=\frac{{\left(c_{1i}c_{2i}\right)}^{0.5}}{{\left(a_{1i}a_{2i}\right)}^{0.5}+{\left(b_{1i}^{1}b_{2i}^{1}\right)}^{0.5}+{\left(b_{1i}^{2}b_{2i}^{2}\right)}^{0.5}+{\left(b_{1i}^{3}b_{2i}^{3}\right)}^{0.5}+{\left(c_{1i}c_{2i}\right)}^{0.5}}, \end{array}$$

Step 5: Assessing the variations of RADR in space and time.

By using the method of rank eigenvalue [38], the assessment grade value *h_i_* of RADR for each evaluation area was calculated by the following equation:

(22) $$h_{i}=a_{i}+2b_{i}^{1}+3b_{i}^{2}+4b_{i}^{3}+5c_{i}$$

when *h_i_* ≤ 1, the assessment grade of RADR belongs to grade I (very strong). When 1 < *h_i_* ≤ 2, it belongs to grade II (strong). When 2 < *h_i_* ≤ 3, it belongs to grade III (moderate). When 3 < *h_i_* ≤ 4, it belongs to grade IV (weak) and when *h_i_* > 4, it belongs to grade V (very weak).

Step 6: Comprehensive assessment of RADR in different evaluation regions for several years.

In this study, the comprehensive assessment of RADR in different regions was analyzed by the improved method of Connection Entropy (CE). {*u* (*i, t*) |*I* = 1, 2, …, *N*; *t* = 1, 2, …, *T*} can describe the RADR for a given region in different years, where *N* and *T* are the total number of evaluation regions and the evaluation years, respectively. According to Equation (19), *u* (*i, t*) is obtained as follows:

(23) $$u(i,t)=a_{it}+b_{it}^{1}I_{1}+b_{it}^{2}I_{2}+b_{it}^{3}I_{3}+c_{it}J$$

the connection components in Equation (22) are the same as those in Equation (8). Then the expression of the CE *S_i_* of *u* (*i*, *t*) is obtained [39]:

(24) $$S_{i}=S_{ia}+S_{ib}^{1}I_{1}+S_{ib}^{2}I_{2}+S_{ib}^{3}I_{3}+S_{ic}J$$

where *I*_1_, *I*_2_, *I*_3_ and *J* are the associated entropy coefficients, and the properties are the same as those in Equation (8). The *S_i_* consists of three parts: Identity entropy *S_ia_*, contrary entropy *S_ic_*, and discrepancy entropy *S_ib_*, in this study, the *S_ib_* was further divided into three parts. The identity, contrary and discrepancy entropy are calculated by the following equations, respectively [40]:

(25) $$S_{i}=\sum_{t=1}^{T}a_{it}\ln\frac{1}{a_{it}}+\sum_{t=1}^{T}b_{it}^{1}\ln\frac{1}{b_{it}^{1}}+\sum_{t=1}^{T}b_{it}^{2}\ln\frac{1}{b_{it}^{2}}+\sum_{t=1}^{T}b_{it}^{3}\ln\frac{1}{b_{it}^{3}}+\sum_{t=1}^{T}c_{it}\ln\frac{1}{c_{it}}$$

where the connection components have been normalized by time is the improvement of CE. Entropy is usually used to describe the degree of disorder or randomness in a system. In the same way, *S_ia_* is a disorder measure, *S_ib_* is an order measure, and *S_ic_* is a chaotic measure of an uncertain system [40]. In this study, the identity and contrary entropy, respectively, reflect the uncertainty of the relationship between RADR and the “strong” or “weak” evaluation criteria. Total entropy *S_i_* reflects the stability of the overall situation of RADR. It evaluates the trend from the perspective of the overall development process, it is also a supplement and improvement to the results of CE evaluation.

Step 7: Diagnosing the vulnerability index of RADR.

The set pair potential based on subtraction was proposed in regional water resources carrying capacity in 2017 [41]. According to the practical application of the diagnostic model of RADR, based on the triple set pair potential based on subtraction in reference, the quintile set pair potential based on subtraction *S_f_* (*u*) was presented:

(26) $$S_{f}(u)=(a_{i}-c_{i})(1+b_{i}^{1}+b_{i}^{2}+b_{i}^{3})$$

Because the value of *S_f_* (*u*) is in the range of from −1 to 1, it is possible to scale the value of the subtraction set and assign the corresponding rank to it. The indexes that had a negative impact on the situation are selected as the vulnerability indexes, and then its set pair potential based on subtraction was compared with the trend of the evaluation grade value, thus not only the original purpose of diagnosing and identifying the different regional vulnerability indexes was achieved. At the same time, the cause of variation and fluctuation of RADR can be analyzed. The calculation of set pair potential based on subtraction reasonably and skillfully avoids the problem of selecting the value of the difference coefficient *I* and the opposites coefficient *J* and does not directly calculate the specific value of the CN but proceeds from the connection component structure of the CN. It provides a scientific basis for the diagnosis and analysis of the vulnerability index of RADR by using the CN.

A total of 30 experts were invited to judge the importance of the five evaluation subsystems and the internal indexes of each subsystem in Table 1 from the actual situation of RADR in the Huaibei Plain. By Equations (2) and (3), six fuzzy complementary judgment matrices were obtained as follows:

(27) $$\begin{array}{l} A=\left[\begin{array}{ccccc} 0.50 & 0.67 & 0.83 & 0.80 & 0.75\\ 0.33 & 0.50 & 0.71 & 0.67 & 0.60\\ 0.17 & 0.29 & 0.50 & 0.44 & 0.38\\ 0.20 & 0.33 & 0.56 & 0.50 & 0.43\\ 0.25 & 0.40 & 0.62 & 0.57 & 0.50 \end{array}\right]\\ A^{1}=\left[\begin{array}{ccc} 0.50 & 0.67 & 0.75\\ 0.33 & 0.50 & 0.60\\ 0.25 & 0.40 & 0.50 \end{array}\right]A^{2}=\left[\begin{array}{ccc} 0.50 & 0.75 & 0.67\\ 0.25 & 0.50 & 0.40\\ 0.33 & 0.60 & 0.50 \end{array}\right]A^{3}=\left[\begin{array}{cc} 0.50 & 0.33\\ 0.67 & 0.50 \end{array}\right]\\ A^{4}=\left[\begin{array}{ccc} 0.50 & 0.67 & 0.75\\ 0.33 & 0.50 & 0.60\\ 0.25 & 0.40 & 0.50 \end{array}\right]A^{5}=\left[\begin{array}{ccc} 0.50 & 0.60 & 0.33\\ 0.40 & 0.50 & 0.25\\ 0.67 & 0.75 & 0.50 \end{array}\right] \end{array}$$

The above fuzzy complementary judgment matrix *A* was substituted as the initial value into Equation (4) and solved by the Accelerated Genetic Algorithm (AGA) AGA, here *d* was 0.2. The weight of each subsystem and the corresponding indexes for evaluating the RADR of the Huaibei Plain in Anhui Province were obtained (Table 2).

## 4. Example of Application

China is located in eastern Asia on the western shore of the Pacific Ocean, affected by special geographical environment and climatic conditions, it has been a country seriously influenced by droughts. With the increasingly prominent contradiction between the water resources supply and demand in China, drought has increased the risk of losses in agricultural production and social economy.

**Figure 1:**
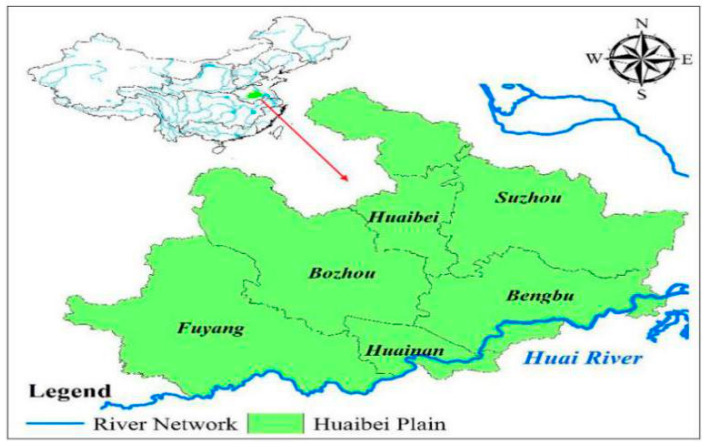
Location of the Huaibei Plain in the Anhui Province.

According to the above assessment and diagnosis method of RADR, the empirical study was conducted in the region of the Huaibei Plain in the Anhui Province. Huaibei Plain (32°45′~34°35′ N, 114°58′~118°10′ E) is located in the north of Anhui province, as shown in Figure 1. The administrative area includes six cities, they are Suzhou, Huaibei, Bozhou, Fuyang, Bengbu, and Huainan.

The Huaibei Plain in the Anhui Province is located in the south of the Cir-cum Huai-Hai Plain. From the perspective of meteorological and hydrological elements, the Huaibei Plain is located in the North-South climate transition zone. The average annual amount of water resources is about 12.87 billion m^3^, and the average amount of water resources per capita is 530 m^3^, which is only half of the average water resources amount per capita in Anhui Province and one fourth of that in China. Obviously, this area belongs to the region where water resources are relatively scarce.

**Figure 2:**
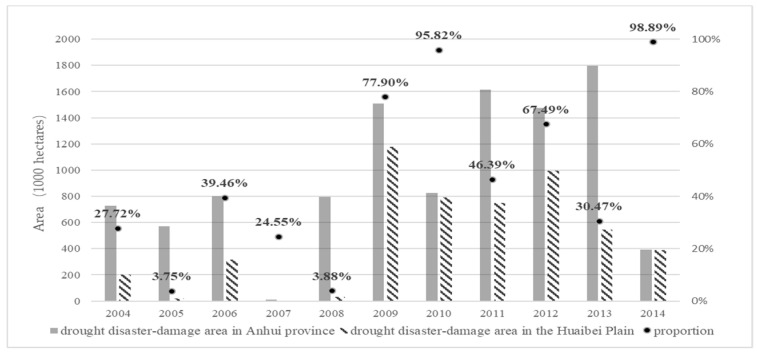
The proportion of the drought disaster-damage areas in the Huaibei Plain accounts for the damage areas in the whole Anhui Province from 2004 to 2014.

From the perspective of agricultural production, the Huaibei Plain is one of the most important agricultural production areas in China, it has a total of 1.80 million hectares of cultivated lands, accounting for 46% of the total cultivated lands of the Anhui Province in 2017. According to the statistics of drought disasters in Anhui Province, due to the special geographical and climatic conditions of the Huaibei Plain, and its important role in agricultural production, the central disaster region of agricultural drought in Anhui Province is located in the Huai River Basin, especially in the Huaibei Plain area. The annual drought disaster-damage area and disaster-affect area in the Huaibei Plain account for 52.5% and 50.8% of the total area of the Anhui Province, respectively (Figure 2). The grain yield reductions induced by droughts reached 48% of the total reductions of the whole province.

In recent years, the Huaibei Plain has experienced a long period of construction to enhance the ability of regional agriculture to resist droughts. In the northern and central regions, water storages are the main projects, water-saving irrigation projects have been improved to ensure water conservation in agriculture. In the southern regions along the Huai River, both sides of the river have been renovated on a certain scale. Rational distribution of water resources has alleviated the situation of water consumption crowding out agricultural water for social and economic development. Therefore, it is necessary to accurately assessment and diagnosis the ability of regional agriculture drought resistance in the Huaibei Plain for guaranteeing national food security.

### 4.1. The Weight of RADRAI in the Huaibei Plain

Based on the characteristic analysis of five evaluation subsystems comprehensively, the situations of the economic and social development in Anhui Province were obtained from the “Anhui Province Statistical Yearbook (2006–2015)”. The natural and social conditions of the water resources were obtained from the main documents, including the “Anhui Province Water Resources Bulletin (2006–2015)”, “Anhui Province Water Resources Statistical Yearbook (2006–2015)”, and “The Statistics of Drought Resistance Planning”. Therefore, the sample values of RADRAI and the corresponding five-levels evaluation criteria in the Huaibei Plain from 2005 to 2014 were obtained (Table 2).

In Table 2, it can be seen that either the human factors in a region of the natural environment or the regional drought adaptability of water conservancy projects had obvious regional differences, which makes them key factors of RADR. Relatively speaking, drought emergency measures can play a vital role in the early stage of drought, but in the later stages, especially in the development of severe drought, the role of emergency measures in drought relief gradually diminishes due to the limited availability of resources. As a result, its importance was slightly lower than the former two. Both the economic and social conditions and the scientific and technological conditions played mainly indirect roles in supporting RADR, but the proportion of government investment in agriculture, forestry, and water reflects the importance of water conservancy construction in the region, thus it also played an important role in many indexes.

### 4.2. The Assessment of RADR in the Huaibei Plain Based on Connection Number

#### 4.2.1. Dynamic Assessment of RADR for Each City in the Huaibei Plain

Firstly, *u*_1*i*_ of RADR for the Huaibei Plain in Anhui Province from 2005 to 2014 were obtained by substituting the weight of each index (Table 2) into Equations (6)–(10), and then *u*_2*i*_ of RADR for the Huaibei Plain in Anhui Province for 10 years was obtained by substituting the sample and weight into Equations (11)–(18). Finally, *u_i_* of RADR from 2005 to 2014 were obtained according to Equations (19) and (20).

Each connection component in the CN represents the degree of correlation between the evaluation object and grades, thus the preliminary evaluation of RADR can be carried out according to the relative size of each connection component in the CN. Assuming that the proportion of *a* in Equation (19) is larger, the relationship between the RADR and the stronger grade is closer, then the RADR is stronger. Otherwise, the greater the proportion of *c*, the weaker the RADR. However, when the distribution of the connection component is more uniform and there is no connection component with a particularly large value, this method has a certain one-jadedness. At this point, the connection component can be used as the initial evaluation grade value when the cumulative value of the connection components is greater than a critical value (this study used 0.67). The relationship between the connection component and the evaluation grade value when the cumulative value of the connection components reached to 0.67 can be seen in Table 3.

**Figure 3:**
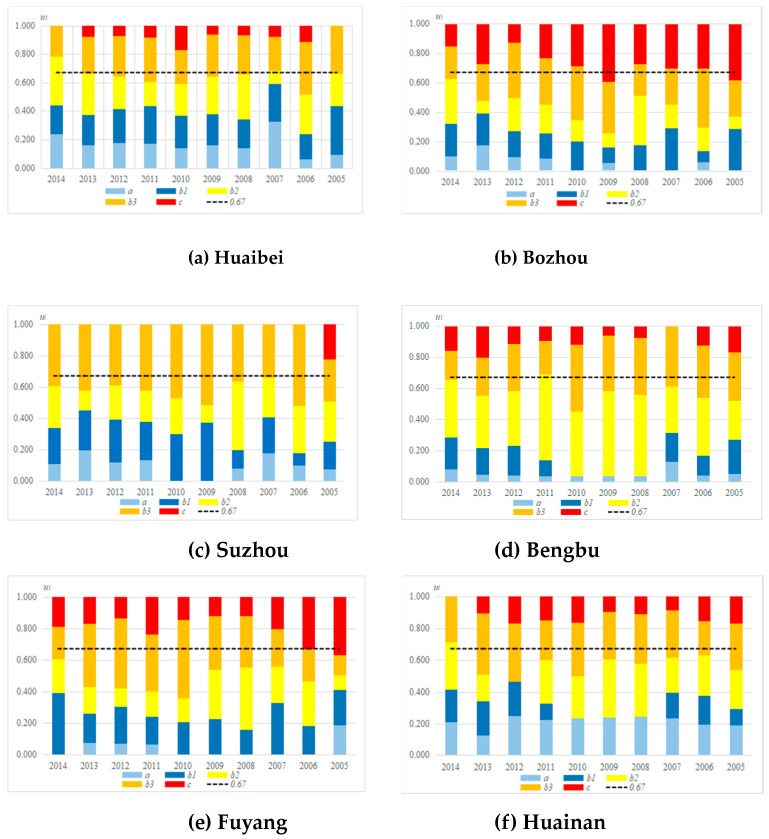
The cumulative value of each connection number *u_i_* of RADR for six cities in the Huaibei Plain from 2005 to 2014.

Therefore, the dynamic assessment of RADR for cities in the Huaibei Plain from 2005 to 2014 was carried out based on the *u_i_* of RADR in Figure 3.

In Figure 3a, from 2005 to 2014, when the cumulative value was greater than or equated to 0.67, the corresponding component was basically *b*2 and *b*3. It is shown that the evaluation grades in Huaibei City have been maintained within the grades III and Ⅳ in the past ten years. The evaluation results in 2014 and 2007 were grade III, which meant that the RADR was relatively strong. From the development trend in the last five years, the proportions of *a*, *b*1, *b*2, *b*3 were relatively stable, but with the continuous decrease of the proportion of *c*, the RADR in Huaibei had been strengthened.

In Figure 3b, from 2005 to 2009, *b*3 accounted for a relatively large proportion in Bozhou, but from 2010 to 2014, the proportion of *b*2 and *a* was increased. It showed that RADR in Bozhou was relatively weak in the Huaibei Plain from 2005 to 2014, especially in 2009 and 2005, it belonged to grade V (very weak). From the perspective of development trend, the RADR had been improved in the past five years. There is a possibility that the RADR will change from weak situation to moderate.

By comparing the Figure 3b,c, it can be seen that the RADR in Suzhou from 2005 to 2014 was similar to the distribution in Bozhou, and the proportion of *b*3 was relatively large. It is shown that although the RADR in Suzhou was in a weak level in the past ten years, there was no worsening trend in drought resilience, thus the possibility of weakened RADR in Suzhou was small.

In Figure 3d, from 2005 to 2014, when the cumulative value of the connection components equaled to 0.67, the corresponding connection component was basically *b*3. It can be seen that in 2008, 2009, and 2010, the evaluation grade values of RADR could be divided into grade IV. In the past five years, with the increase of the proportion of *c*, the RADR gradually approached grade V from 2012 to 2014. The analysis showed that the RADR in Bengbu was weakening on the whole. If the corresponding control measures are not taken in time, the RADR level will be greatly reduced.

In Figure 3e, from 2005 to 2006, the corresponding connection component was basically *c*. As the dominant role of *b*3 in each component had been strengthened, the corresponding component between 2007 and 2014 was basically *b*3. The RADR had been upgraded from grades V to IV, and in the past five years, under the joint effects of the increase of *b*1 and *b*3, the RADR in Fuyang City was still in the process of continuous strengthening, and the development trend is improving.

In Figure 3f, from 2005 to 2014, the proportion of *a* in average five-elements connection number of Huainan City was evenly distributed and always high. However, because the proportions of *b*1 and *b*2 were not high, when the cumulative value of the connection components equaled to 0.67, the corresponding connection component was basically *b*3. The grade was IV, which improved to grade III in 2014. On the one hand, RADR in Huainan had been at a weak level for many years, although it had been strengthened in the past decade but the extent was not large. On the other hand, because of its strong links with stronger grades, its RADR had great potential for development.

In a word, the RADR for six cities in the whole Huaibei Plain mostly belonged to grades III and IV, which was basically consistent with the actual situation of their vulnerability to drought from 2005 to 2014. Among six cities, Bozhou had become a short-board city in the Huaibei Plain. From the development trend, the RADR in Huaibei, Fuyang, and Huainan fluctuated in the past decade, but the overall trends were strengthened, while Bengbu was in a weakening trend, which should be the focus for promoting RADR in the Huaibei Plain.

#### 4.2.2. Spatial and Temporal Distributions of RADR in the Huaibei Plain

**Figure 4:**
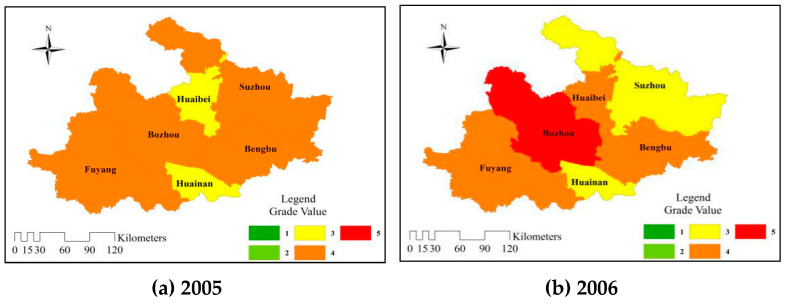

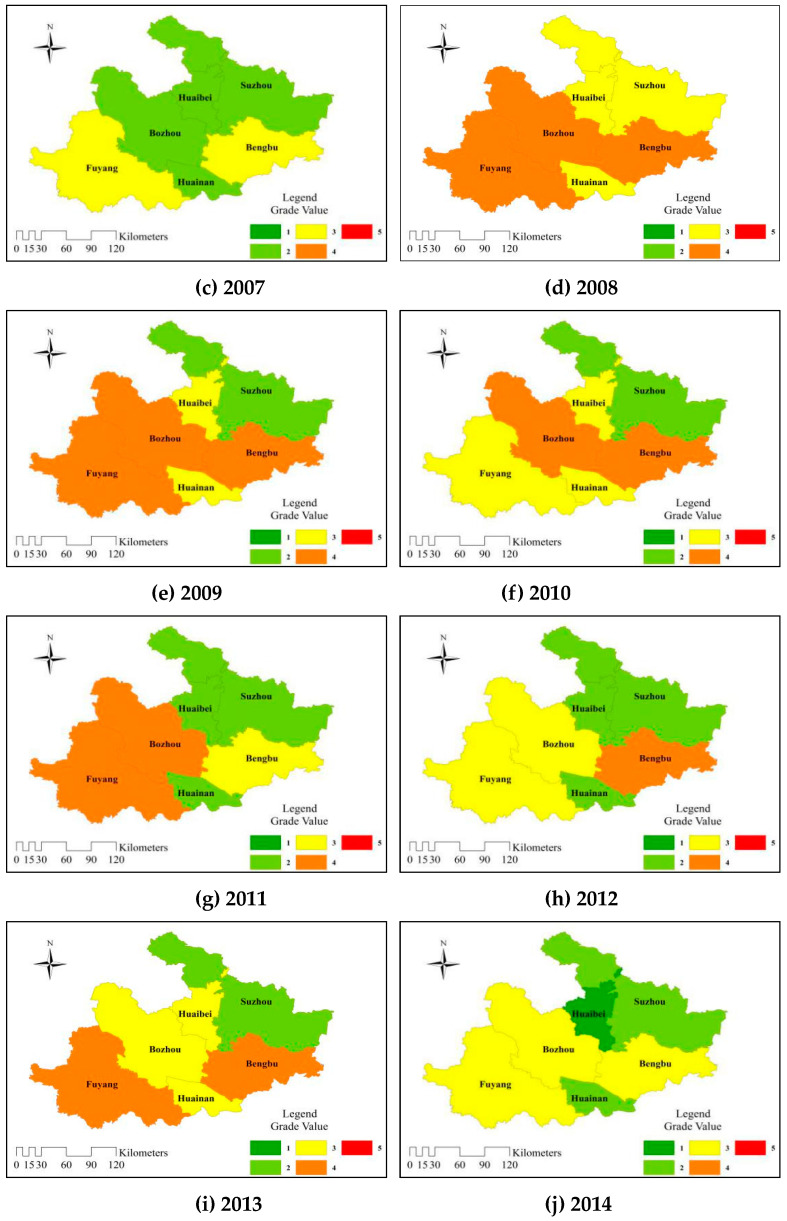
Temporal and spatial distributions of RADR in the Huaibei Plain of Anhui Province.

In order to further analyze the spatial and temporal distributions of RADR for the cities in the Huaibei Plain, the evaluation grades in six cities from 2005 to 2014 were calculated by Equation (21). The evaluation grade value was divided into two parts according to the average method: 1 is close to the grade III (Moderate), 5 is close to the grade IV (Weak). The classification criteria are matched with the corresponding colors as shown in Figure 4.

The differences of RADR between the east and west in the Huaibei Plain were more obvious than those between the north and south. The eastern cities represented by Huaibei, Suzhou, and Bengbu had shown more green areas than the western regions represented by Bozhou, Huainan and Fuyang. In the past five years, the RADR in Suzhou had been at a moderate level, and the fluctuation was smaller than that in the western region, while the RADR in Bozhou had always been weak. In conclusion, the comprehensive analysis showed that the RADR of the whole agriculture in the eastern part was slightly stronger than that in the western part in the Huaibei Plain.

From 2005 to 2009, except for the overall improvement in 2007, the distribution maps were mostly made up of orange and yellow areas. However, in the past five years from 2010 to 2014, there were more green areas, among which Suzhou had been in the green for five consecutive years, and the weak tension in Bozhou had also been alleviated to a certain extent. This showed that although the RADR in the Huaibei Plain was in a moderate and weak level, with the continuous development of the economy and society, the measures taken by local governments to improve the RADR were effective.

#### 4.2.3. Comprehensive Assessment of RADR in the Huaibei Plain Based on Connection Entropy

As can be seen from Table 4, according to the classification criteria described above, the evaluation grade values of each city over several years were basically distributed between grades III (Moderate) and IV (Weak). In Table 4, the minimum evaluation grade value was 2.358 in 2007, and the maximum value was 3.784 in 2009. The average grade value of RADR in the Huaibei Plain from 2005 to 2014 were obtained. The resilience in Suzhou and Huainan for most years were close to each other, which were in grade III (Moderate), and the resilience in Suzhou was the smallest among the six cities. The evaluation level in Bozhou was the largest and the RADR was the weakest among all regions. In summary, the order of RADR from strong to weak in the Huaibei Plain was Suzhou, Huaibei, Huainan, Bengbu, Fuyang, and Bozhou.

**Figure 5:**
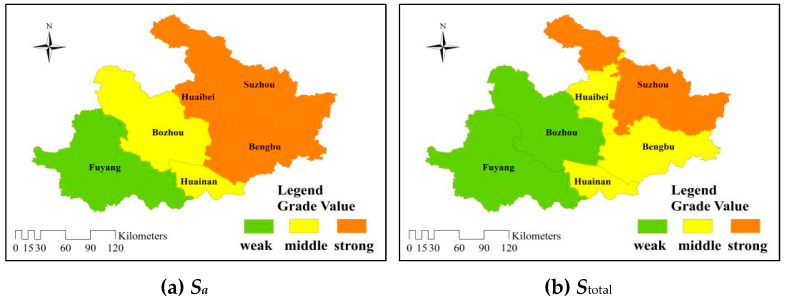
Distributions of entropy value for evaluating the RADR in the Huaibei Plain of Anhui Province. S*_a_* represents the uncertainty of the relationship between the RADR and the “strong” evaluation criteria (grade II); S_total_ reflects the stability of the overall situation of RADR in the future.

In order to comprehensively evaluate the stability and future development trend of RADR in the Huaibei Plain, Equations (23) and (24) were used to calculate the identity entropy *S_a_* of Huaibei, Bozhou, Suzhou, Bengbu, Fuyang and Huainan, and divide them into three levels according to the mean method. The maximum range is shown in red, the smaller is yellow and the smallest is green, as shown in Figure 5a. For the convenience of comparison, the difference coefficient in Equation (23) is 0, and the opposite coefficient is −1. Meanwhile, the total entropy is shown in Figure 5b.

From Figure 5a and Table 5, it can be seen that the identity entropy of six cities in the Huaibei Plain decreased from east to west. The identity entropy values of Huaibei, Suzhou, and Bengbu in the east of were larger, Bozhou and Huainan in the middle were smaller, and Fuyang was the smallest. Because the identity entropy value actually reflects the uncertainty trends in the relationship between the RADR and the “strong” evaluation criteria in the future for each city from 2005 to 2014. The greater the identity entropy value calculated by Equation (21), the better the development situation in the future, the greater the possibility. Therefore, from the point of the identity entropy, the RADR in Huaibei, Suzhou, and Bengbu had higher development potential and are more stable. Similarly, when considering the contrary entropy and discrepancy entropy, it can be seen from Figure 5b that the development potentials and stabilities for six cities in the Huaibei Plain were still decreasing from east to west, while Bengbu and Huainan were weakening. Therefore, the eastern cities like Suzhou and Huaibei not only had a strong ability to cope with drought disasters but also had a higher possibility of ability enhancement.

### 4.3. Diagnostic Analysis of RADR in the Huaibei Plain Based on Set Pair Potential

Based on the comprehensive evaluation of the current situation and development trend of RADR in the Huaibei Plain, this study adopts the set pair potential based on subtraction in the CN, which can not only diagnose and identify the vulnerability index that causes the weakness of RADR in each city but also the causes of inter-annual changes of RADR can be further analyzed from the micro level. The set pair potential based on subtraction *S_f_* (*u*) in the Huaibei Plain from 2005 to 2014 were calculated by Equation (25). The range of *S_f_* (*u*) was divided into five potential levels: They were inverse potential (−1.0 ≤ *S_f_* (*u*) < −0.6), partial inverse potential (−0.6 ≤ *S_f_* (*u*) < −0.2), symmetrical potential (−0.2 ≤ *S_f_* (*u*) ≤ 0.2), partial identical potential (0.2 < *S_f_* (*u*) ≤ 0.6), and identical potential (0.6 < *S_f_* (*u*) ≤ 1.0) [41].

**Figure 6:**
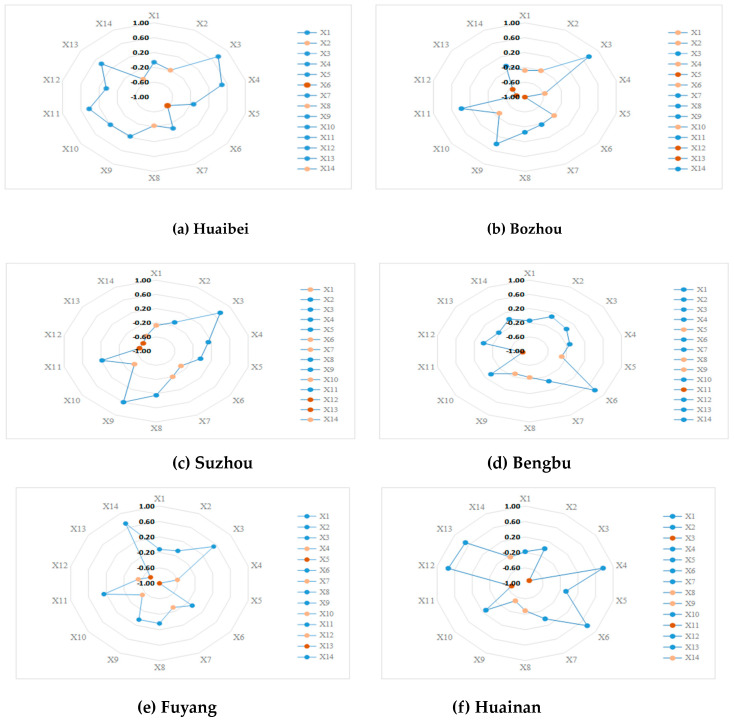
The set pair potential based on subtraction of RADRAI in the Huaibei Plain.

In order to intuitively reflect the distribution of different indexes in each city, the set pair potential is plotted on different radar maps, as shown in Figure 6 below.

In Figure 6, 14 evaluation indexes were classified into three categories. One was the inverse potential index in red, one was the partial inverse potential index in pink, and the other was the ordinary index in blue. Among them, the red and pink markers are the major factors that made the RADR weak, moreover, inverse potential indexes are more disadvantageous than the partial inverse potential indexes.

From the point of different regions, Huaibei had the least number of vulnerability indexes, including one inverse potential index and three partial inverse potential indexes. While Bozhou had the largest number, including three inverse potential indexes and five partial inverse potential indexes. Combined with the assessment results above, it could be found that Suzhou had the strongest RADR in the Huaibei Plain, but the number of its vulnerability indexes was more than that of Huaibei city. Bozhou had the weakest resilience against drought in the Huaibei Plain, which had the largest number of vulnerability indexes. It was shown that there were some deficiencies in all aspects of the RADR in Bozhou, which ultimately resulted in the low level of overall RADR.

From the point of different indexes, the most vulnerable index of RADR in the Huaibei Plain included: X5, X6, X8, X10, X12, X13, and X14. Especially, X14 (agricultural emergency watering capacity per unit area of agricultural area) was regarded as both the inverse potential and the partial inverse potential index in Huaibei, Suzhou, Bengbu, and Huainan. This indicated that agricultural emergency watering capacity was the key vulnerability index of RADR in the Huaibei Plain. Although X13 (rate of drought resilience irrigated land) was only a vulnerability index in Bozhou, Suzhou, and Fuyang, it was the inverse potential index, which meant that it had a greater impact on RADR.

The vulnerability indexes have obvious regional characteristics in Huaibei, Bengbu, and Huainan. X6 (rate of ensure stable yields despite drought or excessive rain) was the main vulnerability index in Huaibei City. According to statistical data, it can be found that the average annual yield of drought and flood protection in Huaibei in the past five years was about 50%, which is lower than the average level of 85% in Anhui Province. In Bengbu, the weak ability of agricultural emergency irrigation was related to the relatively small number of various irrigation and drainage machinery, which can play a key role in alleviating agricultural drought, especially in the early stage of drought. X3 (the percentage of dry land) was the main vulnerability index in Huainan. According to statistical information, the percentage of dry lands in the Huaibei Plain was more than 90%, while in Huainan it was about 35% annually. Although paddy crops are more susceptible to the adverse effects of drought than dry land crops, the planting system in a region is directly related to its climate and geographical conditions, thus it is difficult to change its dry land cultivation areas.

X5 (rate of reservoir storage water) was one of the main vulnerability indexes in Bozhou and Fuyang in the western part. Nowadays, Bozhou and Fuyang use deep groundwater as their water sources for production. They had relatively few small and medium-sized reservoirs to regulate the sustainable relationship between the storage and use of water resources during the drought period. Bengbu and Huainan, located in the southern part, have been in a low level of X11 (rate of water saving irrigation). The small area of water-saving irrigation was the main factor in these two regions. Therefore, the implementation and improvement of water-saving irrigation measures will be key work in the future. X12 (water supply capacity per unit area of agricultural area for drought resilience) of Bozhou and Suzhou in the northern region was weaker than that in other cities, moreover, X13 (rate of drought resilience irrigated land) in Bozhou, Suzhou, and Fuyang remained between 10% and 20%, only about half of Huaibei and Huainan. This indicates that in the northern area, besides Huaibei, targeted management and emergency measures should be adopted to improve the water supply capacity and the intensity of irrigating land in response to droughts.

## 5. Conclusions

In order to quantitatively assess the regional agricultural drought resilience (RADR) and diagnose the vulnerability factors, a model for evaluating and diagnosing the ability of regional agriculture systems to resist drought disasters based on set pair analysis and connection entropy was established. From the application in the Huaibei Plain, the following conclusions were obtained:

There were obvious regional differences between natural and human factors of drought adaptation transformation through water conservancy projects, which were the main factors affecting the RADR. The importance of drought emergency measures was slightly lower because of the limited available water resources restricting the resistance in the late stage of drought, especially during a severe drought. Economic, social, scientific, and technological conditions played indirect supporting roles in regional RADR.

The RADR in the Huaibei Plain from 2005 to 2014 was mainly at a weak level and had a gradually increasing trend. The whole RADR in the Huaibei Plain from 2005 to 2014 was in the order of Suzhou, Huaibei, Huainan, Bengbu, Fuyang and, Bozhou. The average evaluation grade value of RADR in the eastern region (2.887) was smaller than that in the western region (3.141). Therefore, the RADR in the eastern area was slightly stronger than those in the west. The potential stability of RADR development for six cities in the Huaibei Plain was decreasing from east to west. Suzhou had become a city with the most potential, while Fuyang and Bozhou urgently needed to improve the RADR.

The drought emergency condition was the weak link of the RADR in the Huaibei Plain. The natural and water conservancy condition in the eastern region were slightly worse than those in the west. Agricultural emergency watering capacity was the key vulnerability index of RADR in the Huaibei Plain. Drought resilience irrigated land had a greater impact on RADR. The rate of stable yields despite drought was the main vulnerability index in Huaibei City. In Bengbu, the weak ability of agricultural emergency irrigation was related to the small number of various irrigation and drainage machinery. The percentage of dry land was the main vulnerability index in Huainan. Reservoir storage water was one of the main vulnerability indexes in the western part. Bengbu and Huainan, located in the southern part, have been in a low level of water saving irrigation. 

Furthermore, this study suggests that it is urgent to construct the drought emergency facilities and implement the drought resistance measures in the Huaibei Plain. Moreover, the key point to improve the RADR was to focus on the typical cities, which have a weaker ability to resist drought disaster. It can be perceived that for the weakest RADR city, Bozhou should expand the effective irrigation area and improve the irrigation guarantee rate, which could increase the rate of stable yields despite the drought. Fuyang requires the establishment of agricultural water usage, the integration of farmland water conservancy mechanism, and the innovation of farmland water conservancy management for improving the utilization coefficient of agricultural irrigation water. While in Bengbu, the using pressure of agricultural water in the early stages of drought can be alleviated by increasing the number of drainage and irrigation machines.

## Figures and Tables

**Table 1:** The index system for evaluating regional agricultural drought resistance.

Evaluation Subsystem	Evaluation Index		Statistical/Calculated Method
Regional natural condition	X1	Relative Moisture Index (M index)	(Annual rainfall (mm)-Annual evaporation (mm))/Annual evaporation (mm)
	X2	The Amount of Water Resources per Unit Area of Agricultural Area (m^3^/km^2^)	Amount of regional water resources (m^3^)/Agricultural area (km^2^)
	X3	Percentage of Dry Land (%)	Dry land area (km^2^)/Agricultural area (km^2^)
Water conservancy condition	X4	Effective Irrigation Rate of Agricultural Area (%)	Effective irrigation area (km^2^)/Agricultural area (km^2^)
	X5	Rate of Reservoir Storage Water (%)	Regional total reservoir capacity (m^3^)/Surface runoff (m^3^)
	X6	Rate of Stable Yields Despite Drought or Excessive Rain (%)	Stable yields area (km^2^)/Effective irrigation area (km^2^)
Economic and social condition	X7	The Per-capita Net Incomes of Rural Households (yuan)	Statistic caliber
	X8	Proportion of Agriculture and Forestry Water Expenditure (%)	Annual agriculture and forestry water expenditure (yuan)/Annual fiscal expenditure (yuan)
Scientific and technological condition	X9	Water Consumption per Kilogram of Grain (m^3^/kg)	Annual agricultural water consumption (m^3^)/Annual grain output (kg)
	X10	Water Efficiency of Agricultural Irrigation	Statistic caliber
	X11	Rate of Water Saving Irrigation (%)	Water saving irrigation area/Effective irrigation area
Drought emergency condition	X12	Water Supply Capacity per Unit Area of Agricultural Area for Drought Resilience (km^3^/(d·km^2^))	Water supply capacity for drought resilience (km^2^·d)/Agricultural area (km^2^)
	X13	Rate of Drought Resilience Irrigated Land (%)	Drought resilience irrigated land area (km^2^)/Drought affected area (km^2^)
	X14	Agricultural Emergency Watering Capacity per Unit Area of Agricultural Area (per hectare)	Number of irrigation and drainage machinery/Agricultural area (hectare)

**Table 2:** Weights and evaluation grade criteria of regional agricultural drought resilience assessment index (RADRAI) in the Huaibei Plain.

Evaluation Subsystem		Evaluation Index		Evaluation Grade Criterion				
Name	Weight	Name	Weight	I (Very Strong)	II (Strong)	III (Moderate)	IV (Weak)	V (Very Weak)
Regional natural condition	0.2969	X1	0.140	>0.325	(0.102, 0.325]	[−0.102,0.102]	[−0.343, –0.120)	<−0.343
		X2	0.092	>119.56	(87.12, 119.56]	[54.68,87.12]	[22.23, 54.68)	<22.23
		X3	0.065	>98.98	(76.86, 98.98]	[54.75,76.86]	[32.64, 54.75)	<32.64
Water conservancy condition	0.2231	X4	0.105	>91	(81, 91]	[70, 81]	[59, 70)	<59
		X5	0.069	>93.4	(65.6, 93.4]	[37.8, 65.6]	[10.0, 37.8)	<10.0
		X6	0.049	>87	(75, 87]	[63, 75]	[52, 63)	<53
Economic and social condition	0.1369	X7	0.045	>10494	(7724, 10,494]	[4954,7724]	[2184.6, 4954)	<2184.6
		X8	0.092	>14.6	(11.4, 14.6]	[8.3, 11.4]	[5.1, 8.3)	<5.1
Scientific and technological condition	0.1546	X9	0.073	<0.025	(0.025, 0.165]	[0.165,0.305]	[0.305, 0.446)	>0.446
		X10	0.048	>0.530	(0.509, 0.530]	[0.489,0.509]	[0.469, 0.489)	<0.469
		X11	0.033	>98	(67, 98]	[35, 67]	[3, 35)	<3
Drought emergency condition	0.1885	X12	0.089	>52.6	(35.7, 52.6]	[18.7, 35.7]	[1.8, 18.7)	<1.8
		X13	0.042	>57.6	(41.3, 57.6]	[25.1, 41.3]	[8.8, 25.1)	<8.8
		X14	0.058	>0.365	(0.257, 0.365]	[0.148,0.257]	[0.040, 0.148)	<0.040

**Table 3:** The relationship between the connection component and the evaluation grade value when the cumulative value of connection components reaches the critical value.

Connection Component	*a*	*b*1	*b*2	*b*3	*c*
evaluation grade value	I (Very Strong)	II (Strong)	III (Moderate)	IV (Weak)	V (Very Weak)

**Table 4:** Evaluation grade values of RADR in the Huaibei Plain of Anhui Province.

Year	City in the Huaibei Plain of Anhui Province						Average
	Huaibei	Bozhou	Suzhou	Bengbu	Fuyang	Huainan	
2014	2.386	3.068	2.745	3.098	2.942	2.502	2.790
2013	2.810	3.197	2.435	3.348	3.283	3.045	3.020
2012	2.777	3.154	2.644	3.241	3.046	2.621	2.914
2011	2.775	3.428	2.673	3.033	3.480	2.886	3.046
2010	3.025	3.538	2.685	3.360	3.188	2.880	3.113
2009	2.833	3.784	2.453	3.114	3.188	2.664	3.006
2008	2.902	3.484	2.861	3.080	3.229	2.639	3.032
2007	2.358	3.298	2.537	2.729	3.114	2.723	2.793
2006	3.249	3.764	2.913	3.320	3.510	2.915	3.278
2005	2.542	3.414	3.378	3.307	3.232	2.988	3.144
Average	2.766	3.413	2.732	3.163	3.221	2.786	3.041

**Table 5:** The identity entropy and total entropy for six cities in the Huaibei Plain from east to west.

City	Suzhou	Huaibei	Bengbu	Huainan	Bozhou	Fuyang
	East 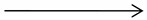 West					
identity entropy *S_a_*	2.032	2.222	2.201	1.856	1.713	1.264
total entropy *S*_total_	1.032	0.510	0.400	0.426	0.230	0.112

## References

[1] Loon A.F.V., Gleeson T., Clark J., Van A.I.J.M., Stahl K., Hannaford J. (2016). Drought in the Anthropocene. Nat. Geosci..

[2] Pritchard H.D. (2017). Addendum: Editorial Expression of Concern: Asia’s glaciers are a regionally important buffer against drought. Nature.

[3] Chaves M.M., Maroco J.P., Pereira J.S. (2003). Understanding plant responses to drought—From genes to the whole plant. Funct. Plant Biol..

[4] Tan M.L., Tan K.C., Chua V.P. (2017). Evaluation of TRMM product for monitoring drought in the Kelantan River Basin, Malaysia. Water.

[5] Carr E.R., Kettle N.P. (2009). Commentary: The challenge of quantifying susceptibility to drought-related crisis. Reg. Environ. Chang..

[6] Pedro-Monzonís M., Solera A., Ferrer J. (2015). A review of water scarcity and drought indexes in water resources planning and management. J. Hydrol..

[7] Schwalm C.R., Anderegg W.R.L., Michalak A.M., Fisher J.B., Biondi F., Koch G. (2017). Global patterns of drought recovery. Nature.

[8] Jarraud M., Steiner A. (2012). Managing the risks of extreme events and disasters to advance climate change adaptation: Foreword. J. Clin. Endocrinol. Metab..

[9] Zhao M.S., Running S.W. (2010). Drought-induced reduction in global terrestrial net primary production from 2000 through 2009. Science.

[10] Mishra A.K., Singh V.P. (2010). A review of drought concepts. J. Hydrol..

[11] Brusberg M.D., Shively R. (2015). Building drought resilience in agriculture: Partnerships and public outreach. Weather Clim. Extrem..

[12] Medd W., Chappells H. (2007). Drought, demand and the scale of resilience: Challenges for interdisciplinarity in practice. Interdiscip. Sci. Rev..

[13] Mitchell G., Mcdonald A. (2015). Developing resilience to England’s future droughts: Time for cap and trade?. J. Environ. Manag..

[14] Wang F., Zhang G., Han Y. (2013). Research on social-ecological system’s drought resilience in Henan Province. Water Resour. Power.

[15] Sawada Y., Koike T. (2016). Ecosystem resilience to the Millennium drought in southeast Australia (2001–2009). J. Geophys. Res. Biogeosci..

[16] Oliver T.H., Brereton T., Roy D.B. (2013). Population resilience to an extreme drought is influenced by habitat area and fragmentation in the local landscape. Ecography.

[17] Merritt W.S., Patch B., Reddy V.R., Syme G.J. (2016). Modelling livelihoods and household resilience to droughts using Bayesian networks. Environ. Dev. Sustain..

[18] Ledger M.E., Harris R.M.L., Armitage P.D. (2012). Climate change impacts on community resilience: Evidence from a drought disturbance experiment. Adv. Ecol. Res..

[19] Du T.L.T., Bui D.D., Buurman J., Quach X.T. (2018). Towards adaptive governance for urban drought resilience: The case of Da Nang, Vietnam. Int. J. Water Resour. Dev..

[20] Vetter S. (2009). Drought, change and resilience in South Africa’s arid and semi-arid rangelands. S. Afr. J. Sci..

[21] Sun Y.H., Zhou H.J., Jing A.W., Yuan Y. (2012). Farmers’ response to agricultural drought in paddy filed of southern China: A case study of temporal dimensions of resilience. Nat. Hazards.

[22] Rockström J. (2003). Resilience building and water demand management for drought mitigation. Phys. Chem. Earth.

[23] Rey D., Holman I.P., Knox J.W. (2017). Developing drought resilience in irrigated agriculture in the face of increasing water scarcity. Reg. Gov. Chang..

[24] Falkenmark M., Rockström J. (2008). Building resilience to drought in desertification-prone savannas in Sub-Saharan Africa: The water perspective. Nat. Resour. Forum.

[25] Gu Y., Ni S.H., Wang H.R. (2005). Comprehensive evolution on ability of coping with agriculture drought in China. Adv. Water Sci..

[26] Cho J., Ko G., Kim K. (2016). Climate change impacts on agricultural drought with consideration of uncertainty in CMIP5 scenarios. Irrig. Drain..

[27] Hao Z., Singh V.P. (2015). Drought characterization from a multivariate perspective: A review. J. Hydrol..

[28] Fei Z.Y., Zhou Y.L., Jin J.L. (2013). Construction of evaluation index system and model for regional drought resistance ability. J. Catastrophol..

[29] Smit B., Burton I., Klein R.B., Street R. (1999). The science of adaptation: A framework for assessment. Mitig. Adapt. Strateg. Glob. Chang..

[30] Liang Z.M., Li J.Q., Chang W.J. (2013). Research on theoretical framework of drought resistance ability. South North Water Transf. Water Sci. Technol..

[31] Jin J.L., Fei Z.Y., Li J.Q. (2013). Method of evaluating the regional drought resistance ability based on the analysis of water balance between supply and demand withe different water frequencies. J. Hydraul. Eng..

[32] Jin J.L., Wu K.Y., Li R.Z. (2007). Region water security evaluation method based on information entropy and improved fuzzy analytic hierarchy process. J. Hydroelectr. Eng..

[33] Lu Y.J. (2002). Weight calculation method of fuzzy analytical hierarchy process. Fuzzy Syst. Math..

[34] Song G.X., Yang D.L. (2003). Methods for identifying and improving the consistency of fuzzy judgment matrix. Syst. Eng..

[35] Kumar K., Garg H. (2016). TOPSIS method based on the connection number of set pair analysis under interval-valued intuitionistic fuzzy set environment. Comput. Appl. Math..

[36] Wang W.S., Jin J.L., Ding J., Li Y.Q. (2009). A new approach to water resources system assessment—Set pair analysis method. Sci. China Ser. E Technol. Sci..

[37] Neri C., Schneider L. (2014). The impact of the prior density on a minimum relative entropy density: A case study with SPX option data. Entropy.

[38] Zou Q., Zhou J.Z., Zhou C., Chen S.S. (2012). Flood disaster risk analysis based on principle of maximum entropy and attribute interval recognition theory. Adv. Water Sci..

[39] Zhao K.Q. (2000). Set Pair Analysis and Preliminary Application.

[40] Pan Z.W., Jin J.L., Li C.H., Ning S.W., Zhou R.X. (2017). A connection entropy approach to water resources vulnerability analysis in a changing environment. Entropy.

[41] Cui Y., Feng P., Jin J.L., Liu L. (2018). Water resources carrying capacity evaluation and diagnosis based on set pair analysis and improved the entropy weight method. Entropy.

